# Artificial intelligence feasibility in veterinary medicine: A systematic review

**DOI:** 10.14202/vetworld.2023.2143-2149

**Published:** 2023-10-21

**Authors:** Fayssal Bouchemla, Sergey Vladimirovich Akchurin, Irina Vladimirovna Akchurina, Georgiy Petrovitch Dyulger, Evgenia Sergeevna Latynina, Anastasia Vladimirovna Grecheneva

**Affiliations:** 1 Department of Animal Disease, Veterinarian and Sanitarian Expertise, Faculty of Veterinary Medicine, Vavilov Saratov State University of Genetic, Biotechnology and Engineering Saratov, Russia; 2 Department of Veterinary Medicine, Russian State Agrarian University- Moscow Agricultural Academy named after K.A. Timiryazev, 49, str. Timiryazevskaya, Moscow, 127550, Russia; 3 Department of Applied Informatics, Russian State Agrarian University-Moscow Agricultural Academy named after K.A. Timiryazev, 49, str. Timiryazevskaya, Moscow, 127550, Russia

**Keywords:** artificial intelligence, Cochrane study, criterion, extracted data, heterogeneity, systematic review

## Abstract

**Background and Aim:**

In recent years, artificial intelligence (AI) has become increasingly necessary in the life sciences, particularly medicine and healthcare. This study aimed to systematically review the literature and critically analyze multiple databases on the use of AI in veterinary medicine to assess its challenges. We aim to foster an understanding of the effects that can be approached and applied for professional awareness.

**Materials and Methods:**

This study used multiple electronic databases with information on applied AI in veterinary medicine based on the current guidelines outlined in PRISMA and Cochrane for systematic review. The electronic databases PubMed, Embase, Google Scholar, Cochrane Library, and Elsevier were thoroughly screened through March 22, 2023. The study design was carefully chosen to emphasize evidence quality and population heterogeneity.

**Results:**

A total of 385 of the 883 citations initially obtained were thoroughly reviewed. There were four main areas that AI addressed; the first was diagnostic issues, the second was education, animal production, and epidemiology, the third was animal health and welfare, pathology, and microbiology, and the last was all other categories. The quality assessment of the included studies found that they varied in their relative quality and risk of bias. However, AI aftereffect-linked algorithms have raised criticism of their generated conclusions.

**Conclusion:**

Quality assessment noted areas of AI outperformance, but there was criticism of its performance as well. It is recommended that the extent of AI in veterinary medicine should be increased, but it should not take over the profession. The concept of ambient clinical intelligence is adaptive, sensitive, and responsive to the digital environment and may be attractive to veterinary professionals as a means of lowering the fear of automating veterinary medicine. Future studies should focus on an AI model with flexible data input, which can be expanded by clinicians/users to maximize their interaction with good algorithms and reduce any errors generated by the process.

## Introduction

Medicine has always been and will likely remain an *averag*e profession, wherein offered treatments correspond to the most effective plan for the *average* patient. Individual variation might negate this assumption, and as a result, false positive and false negative results might arise. The more the treatment process can be digitalized, the more precise outcomes can become. In recent years, artificial intelligence (AI) has become increasingly necessary in the life sciences, particularly in medicine and healthcare.

Chang [1] reviewed the main areas of AI focus, which included advantages for imaging interpretation using deep-machine learning (ML), which can help with decision-making, digitalization, which can aid in administrative support and natural language processing for communication, and education and training, which can be used for data mining, risk assessment, and prediction.

Artificial intelligence has been widely adopted and applied in veterinary medicine to improve animals’ healthcare by maximizing predictive indicators and achieving greater accuracy in diagnosis. Machine learning interacts with imaging, pathology slides, and patients’ electronic medical records to aid in reaching the correct diagnosis, prescribing appropriate therapy, and augmenting professionals’ capabilities [2].

Several areas have attempted to improve diagnosis and disease control through the application of AI. Laboratory hematology analyzers and imaging machines include AI expert systems, and mathematical algorithms use raw input data to provide clinical interpretation [3]. At present, there are growing concerns regarding the comparison of clinicians to AI algorithms, and to what extent do AI outcomes support an accurate clinical decision. For instance, based on slide scanning, digital pathology is more accurate than humans evaluating high-resolution slides. However, veterinarians link these AI findings to the patient’s clinical background before making further decisions. Artificial intelligence tools developed for this field have a diagnostic accuracy of up to 95% and are almost 100 times faster in providing results [4, 5]. In this study, we intend to qualitatively and quantitatively describe the current state of applied AI in veterinary medicine, elucidate future trends, and critically interpret outcomes of those fields that have applied Hi-Tech methods.

To the best of our knowledge, there has been no published systematic literature review on the use of AI in veterinary medicine. Therefore, this study aimed to review and critically analyze the literature in different databases and offer a qualitative assessment of these findings with a descriptive analysis of applied AI in veterinary medicine.

## Materials and Methods

### Ethical approval

Ethical Committee approval was not required because the study was based on a systematic review.

### Study period and location

The electronic databases PubMed, Embase, Google Scholar, and Scopus were thoroughly screened up to March 22, 2023, for the use of AI in veterinary medicine. The data were extracted at Department of Veterinary Medicine, Russian State Agrarian University, Moscow.

### Search strategy, selection criteria, and study selection

**Figure-1:**
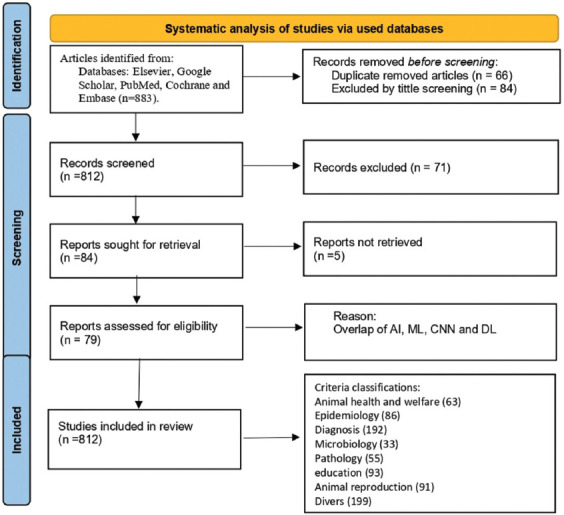
Flowchart of systematic review process.

The search strategy was developed based on our previous studies and was modified based on the co-authors’ views. Article screening was conducted according to the most up-to-date guidelines for systematic reports and meta-analysis as outlined in PRISMA [6] and Cochrane [7]. The electronic databases PubMed, Embase, Google Scholar, and Scopus were thoroughly screened through March 22, 2023, for the use of AI in veterinary medicine. The keywords were terms relevant to animal species, veterinary medicine, and AI. All screenings were performed based on the publication title or the abstract if the full-text was unavailable. Identified citations were imported into Endo-file. The approach process and identification of the reviewed articles are illustrated in the flow diagram (Figure-1).

A population, intervention, control, and outcomes (PICO) note on external validity was attached to each citation. The study design was carefully chosen to provide quality evidence, as randomized trials without significant limitations provide high-quality and stronger evidence. No automatic filtration was applied in this search, and other references used for the search are listed in the appropriate part of the study. All aspects of AI, including ML, convolutional neural network (CNN), and deep learning (DL) were accepted as part of the search results. A total of 79 relevant studies were retrieved from the search criteria and were included in this study.

### Extracted/included data

For a study to be included in the search, it had to have been an original research publication in a peer-reviewed journal, conference, or book accessible to the reviewers. There were no limitations regarding either country or language of origin of the study. The publication have to describe the use of AI in veterinary medicine. There were no restrictions on study design, and randomized or non-randomized controlled trials, interventional, observational, or case studies were included. Failure to comply with the required criteria resulted in the exclusion of the publication. In addition, any duplicated publications were excluded from the study.

Which category to assign each study to, as noted in Table-1, was discussed and agreed on by all co-authors. The authors developed a table containing 19 criteria specifically for use in extracting data from papers on the use of AI per individual specialty in the veterinary profession.

**Table-1:** Standardized conceptions for studies categorization.

Category number	Category nomination	Category description
1	Microbiology	All articles related to bacteriology, virology, and mycology.
2	Diagnostic	Imaging, MRI, CT scanning, and different testing.
3	Epidemiology	Infectious diseases (spreading, distribution, and surveillance), risk factors, monitoring systems, prevention, and forecasting.
4	*Animal Health and welfare*	Treatment, drugs, surgeries, ethics, and animal well-being.
5	*Education*	e-learning, administrative support, and teaching process.
6	*(Digital) pathology*	Different pathogenic agents, pathogenicity, histopathology, and physiopathology.
7	*Animal reproduction*	Farming management breeding of all species.
8–19	*Divers*	Includes toxicology, pharmacology, oncology, hematology, anatomy, nutrition, anesthesia, statistics, biochemistry, histology, embryology, and ecology.

MRI=Magnetic resonance imaging, CT=Computed tomography

It was agreed that a minimum of 30 reports were necessary to define a specific category. Any criterion with fewer than 30 studies was classified as part of the diverse category. This threshold significantly decreased the number of different criteria and maximized the study’s ability to focus on critical AI orientation. Reviews and references obtained through the search were equally and randomly distributed to all co-authors, who individually screened them based on the study design criteria. There was a total of 19 articles that could have been attributed to more than one criterion. In this situation, all reviewers discussed their views until a consensus was reached. Two independent reviewers checked the accuracy of the selection process by reviewing a sample of the included studies, and they agreed with the PICO characteristics of the studies.

### Exclusion criteria

The publications with AI use but not related to veterinary medicine were excluded. Failure to comply with the required criteria resulted in the exclusion of the publication. In addition, any duplicated publications were excluded from the study.

### Statistical framework of heterogeneity

Cochran’s Q test is the traditional test for heterogeneity in meta-analyses. This allows for the idealization of the study size within a desired level of precision.

Cochran formula:


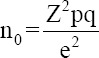


In the equation, e is the level of precision; p is the proportion of the population; q = 1 − p; and the value of Z is given in Z-table [7, 8].

To set a target population, we need to determine how many AI articles must be reviewed to have maximum variability (randomized trial). Using a 95% confidence limit and p = 0.05, we found that a random sample of 385 articles in our target population should be sufficient to give the desired confidence levels.

### Quality assessment (risk of bias)

The scientific quality of each article was assessed according to the Cochrane guidelines [7]. The result of the assessment is noted in Table-2. A standardized form containing nine criteria was developed to assess each paper’s overall quality and risk of bias. The quality rating scale contained nine items and were rated by two independent reviewers on a binomial scale and was summed to give an overall indication of quality [7].

**Table-2:** Quality assessment of the systematic review (Risk of Bias).

Items number	Description of rating scale	Score
1	Definition of AI essence	
	Clear definition of AI used, for example, measuring Glucose, scanning for diagnosis, digital pathology, smears/slides readers…	1
	subtle AI definition, for example, comparison between clinician and AI accuracy	0
2	Source of data	
	Peer-reviews from the above databases	1
	Additional references	0
3	Study length	
	>10 years	1
	≤10 years	0
4	Clearance of duration	
	Defined period like, from 2017 to 2023	1
	Unclear study period, for instance, 10-year period or for the last decade…	0
5	Study population	
	No. of animals, cases, and outbreaks were reported	1
	Losses, expenses, successful cases or not were reported rather than epidemiological parameters	0
6	AI efficacy comparisons	
	Comparisons were made between AI outperformance and professionals/clinicians	1
	No comparison	0
7	Appropriateness of error-generating	
	AI algorithms generating outcomes are not adjusted by clinicians	1
	AI algorithms generating outcomes are not free-error	0
8	Statistics importance	
	*P*and *CI*were reported	1
	*P*and *CI*were not reported	0
9	Study limitations	
	Reviewers identified a possible existence of bias	1
	Risk of bias was infinitesimal to none	0

CI=Confidence interval

## Results

### Overview

The initial electronic search identified 883 references. After screening and selection, 812 studies were included in the study and 71 were excluded from the study. Based on the analysis noted above, a random sample of 385 of the 812 identified articles should be sufficient to give the study a 95% confidence level.

**Figure-2:**
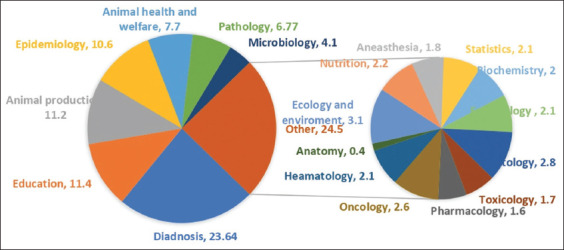
Artificial intelligence allotment percentage in veterinary sector as per databases (PubMed, Embase, Google Scholar, and Scopus) screening up to March 22, 2023.

There were four major areas that encompassed AI implications in the veterinary field. The first area was diagnostic studies. The second area was papers involving education, animal production, and epidemiology with an equal number of papers on each topic. The third area included animal health and welfare, pathology, microbiology, and duplicated citations with an approximately equal number of papers on each of these topics. The remaining area consisted of papers in all other subcategories that did not reach the threshold of 30 papers each. A detailed scheme of this breakdown of topics and areas is noted in Figure-2.

### Systematic search

In fact, 150 articles were eventually excluded following further retrospective analysis. A total of 79 citations were retrieved based on their full-text analysis.

### Descriptive analysis

Figure-1 summarizes the included characteristics of the this study. The 812 works were published up to March 22, 2023, and these studies were conducted worldwide. A total of 192 studies were categorized as diagnostic, 93 as education, 91 as animal production, 86 as epidemiology, 63 as animal health and welfare, 55 as pathology, and 33 as microbiology. As noted above, the remaining studies did not qualify for a separate category. These remaining diverse studies included toxicology, 14; pharmacology, 13; oncology, 21; hematology, 17; anatomy, 3; nutrition, 18; anesthesia, 15; statistics, 17; environment and ecology, 25; biochemistry, 16; histology, 23; and embryology, 17.

In addition, 79 citations were added to the included data after a full-text review of the search results found that AI was also referred to by the terms ML, CNN, and DL. Machine learning, CNN, and DL were referred to 36.7%, 16.45%, and 46.83% of the time, respectively.

### Characteristics of the included studies

During the course of reviewing the selected articles, their categorization could change. For instance, studies related to hematology covered a wide range of diseases and blood abnormalities such as blood cancer, blood analysis, and hereditary or genetic diseases. The classification of these topics changed as we removed studies related to cancer from hematology and added them to oncology after all co-authors agreed to such a move. All articles were given a category such as epidemiology or diagnosis and a subcategory such as blood cancer, risk factors, infectious diseases, or metabolism. Subsequently, a final classification was made based on unifying subfields in the same category.

### Category selection

The documents were classified as peer-reviewed studies, journal/book papers, or conference reports during the collection and review process. Particular attention was paid to peer-reviewed studies (65%), as they detailed their research process as opposed to conference reports (10%), which focused more on results. Table-3 categorizes all of the collected works.

**Table-3:** Categorization of obtained studies (using AI in veterinary medicine) from databases PubMed, Embase, Google Scholar, and Scopus up to March 22, 2023.

Study type	Number of articles
Peer-reviews studies	579
Conferences reports	89
Journals/books papers	215

A meta-analysis of these studies was not possible due to the scarcity and lack of raw data required to determine accepted accuracy measures, but also due to the inability to combine the various criteria in this approach. In addition, comments on the illustrated figures of pooled metrics were not produced. The same reason has been documented in similar systematic reviews of AI applications [9, 10].

### Quality assessment

Systematic Cochrane reviews need to be combined with minimal systematic error, also known as bias, to provide outcomes with a level of credibility [11]. To assess the quality of this study, a standardized table containing nine criteria was developed to assess the overall quality and risk of bias associated with each paper. The quality rating scale contained nine items. They were rated by two independent reviewers on a binomial scale and summed to give an overall indication of quality [7, 11].

The score rating intervals are as follows:

- 1^st^ interval: 0–2, relatively low quality
- 2^nd^ interval: 3–6, moderate (acceptable) quality, and
- 3^rd^ interval: 7–9, relatively high quality.

File standardization resulted in nine agreed criteria, which were attributed to reviews. There are items, such as the definition of AI essence, where the data source was highly biased due to the study design. The appropriateness of error generation was assessed based on staff intervention to agree, adjust, or add another control test. This is a critical process in delivering a reliable but accurate medical decision. However, comparing AI efficacy to clinicians’ performance has no risk of bias, which leads us to conclude that completeness is the key feature of this study, not comparison. Other components of the standardized table (study length, population, limitations, and statistical approach) show zero to very low evidence of bias, meaning that the outcomes of this study are trustworthy (3^rd^ interval, acceptable to high).

Only three items from the quality assessment were rated as biased: The definition of AI essence, the data source, and the appropriateness of error generation. A score of three indicates a likelihood that data on applied AI was biased, and the ultimate outcome appears to have a low to moderate risk of bias (second interval). The quality assessment of the included studies varied in their relative quality and risk of bias. The findings of the six items that scored six out of a total of nine points on the quality scale are more robust regarding the accuracy of AI outperformance and the extent of AI in the veterinary profession.

## Discussion

Following the current review, the data on applied AI in the veterinary sphere were assigned to seven categories, and the uncategorized citations were grouped under a diverse category. The minimum threshold criterion noted above aided in minimizing quality assessment errors.

The diagnostic process is greatly influenced by the latest AI advancements, which lead to a shorter time to diagnosis and more confidence that an appropriate decision was made. Several practical settings benefit from AI, such as atrial fibrillation detection, seizures, hypoglycemia, and the diagnosis of several diseases [12]. Clinicians have acquired many AI applications to aid in diagnosing, supervising, and monitoring diseases successfully in daily practice. However, clinical precision is still questionable, and the appropriateness of a generating error was biased (Table-2). This is due to outcomes being derived from generating algorithms of pre-existing data. The possible occurrence of marginal error and limitations is linked to the design model of AI, which is also called the overfitting phenomenon [13, 14].

The reliability of AI application has been discussed in different domains, and the behavior of many professionals toward its outcomes is due to evidence of questionable efficiency compared to clinicians. Artificial intelligence can produce unreliable outcomes due to lack of primary data replication and built algorithms overlapping from one case to another [12, 15].

Future studies should focus on an AI model that has flexible input data. This model could be expanded by clinicians to maximize their interaction without altering outperforming algorithms and alleviating generated errors.

Another key area is AI-derived applications that focus on revealing and confirming clinical opinions already initiated by a veterinarian. In this context, the clinicians’ reluctance to embrace advances in AI may be problematic unless the technology supports the clinician’s opinion.

Educational use represents 11.4% of total usage for administrative and linguistic support, data mining, learning process, communication, and research. Epidemiological purposes (10.56%) can aid with risk factor assessment, forecasting and prevention, surveillance programs, and other assessments for envisioning potential future strategies.

The challenges of AI have been addressed intensively in veterinary medicine, with 92% of the retrieved studies published in the past 3 years. However, AI outcomes should not be relied upon exclusively as they might be incomplete, heterogeneous, erroneous, or inaccurate. Bologna and Hayashi noted that the best-performing methods are often the least transparent, and those providing a clear explanation (e.g., decision trees) are less accurate [16]. There are concerns about limited human control in the human-AI relationship and the AI-produced interpretation might lead to inaccurate decision-making. In this instance, the explainability and causality of AI are crucially useful and can promote acceptance using human guidance if needed [17].

The criticized key patterns in the current systematic review should be applied to AI doctors (medical chatbot, augmented doctors, and medical curricula) that might have been awaited by the public but not by cutting-edge veterinary practitioners. The concept of ambient clinical intelligence seems to be adaptive, sensitive, and responsive to the digital environment and may be attractive to professionals as a means of lowering the fear of automating veterinary medicine [18, 19]. Finally, in the critical process of delivering a reliable and accurate medical decision, professional veterinary skepticism must lead the process as completeness and accuracy is the key feature, not a comparison between humans and AI. Artificial intelligence can drive the process, but veterinarians are required to guide it.

The quality outcomes of this study were assessed from a random sample (n = 385) of the 883 articles obtained in our systematic review. The recommended review quality stops at 145 at p = 0.05. We reviewed 385 articles to have minimal systematic error. The risk of bias was valued binomially, and the review authors established a list of nine criteria to be assessed. The total risk of bias was low to moderate, which validated the quality of the current approach.

## Conclusion

AI has significant implications in the following areas in veterinary medicine: First, diagnostics; second, education, animal production, and epidemiology; third, animal health and welfare, pathology, and microbiology; and fourth, all remaining categories. Assessment of the appropriateness of error-generating and AI efficacy led us to conclude that AI-derived answers should be used to enhance veterinary ability, not compared to it. The concept of ambient clinical intelligence seems to be adaptive, sensitive, and responsive to the digital environment and may be attractive to veterinary professionals as a means of lowering the fear of automating veterinary medicine.

Future studies should focus on an AI model with flexible data input, which can be expanded by clinicians/users to maximize their interaction with good algorithms and reduce any errors generated by the process. We recommend that the extent of AI in veterinary medicine should be increased, but it should not take over the profession.

## Authors’ Contributions

ASV: Proposed the concept of the research, BF and AIV: Developed the search approach. DGP, LES, and GAV: Collected data. BF and DGP: Classified and categorized the data. LES and GAV: Drafted and revised the manuscript. All authors participated in data collection and elaboration process. All authors have read, reviewed, and approved the final manuscript.
